# Transforming Healthcare: The Revolutionary Benefits of Cashless Healthcare Services

**DOI:** 10.7759/cureus.50971

**Published:** 2023-12-22

**Authors:** Nikhil Sagare, Nandkishor J Bankar, Shivani Shahu, Gulshan R Bandre

**Affiliations:** 1 Hospital Administration, School of Allied Health Sciences, Datta Meghe Institute of Higher Education and Research, Nagpur, IND; 2 Microbiology, Jawaharlal Nehru Medical College, Datta Meghe Institute of Higher Education and Research, Wardha, IND

**Keywords:** digital transactions, cashless economy, improved patient tracking, reduced administrative burden, security, cashless hospital services, transforming healthcare

## Abstract

As cashless hospital services have grown in popularity, the healthcare sector has seen a tremendous transition. That means the payments are done in an electronic manner which is also known as e-pay. The article discusses the benefits of cashless healthcare services, which are transforming the healthcare sector by providing a streamlined, secure, and effective experience for patients and healthcare providers. Cashless healthcare facilities make use of cutting-edge technologies, including mobile applications, digital wallets, and secure internet platforms, to optimize the utilization of resources within the healthcare ecosystem and improve the overall patient experience. The incorporation of technology has led to revolutionary innovations that continue to redefine the way people access and experience health services. The advantages of cashless hospital services have transformed the healthcare sector by enhancing data security, facilitating transparent billing, and encouraging a patient-centered approach. Cashless services are a preferred method of payment for both consumers and organizations due to their convenience and accessibility. Patients can make payments using digital channels such as mobile payment applications, online payment gateways, or contactless payment cards, whether they are paying for medications, lab tests, or complicated surgeries. Cashless transactions drastically reduce administrative challenges for healthcare providers by eliminating the requirement for manual documentation, which facilitates quick electronic transactions and reduces processing times. As the billing and payment process becomes digitized and streamlined, doctors and medical personnel can focus more on treating and caring for patients. Additionally, much faster insurance claim processing and verification processes result in quicker pay-outs and minimize the financial burden on patients.

## Introduction and background

In the rapid development of modern healthcare, convenience and innovation have taken the lead in transforming medical services [1,2]. "Cashless healthcare services" is one such influential idea that is transforming the sector and empowering patients all over the world. By eliminating traditional inconveniences related to payment methods, this revolutionary approach to healthcare provides a streamlined, secure, and effective experience for patients and healthcare providers [3,4]. Healthcare providers that accept cashless payments mark a significant change from earlier payment methods that relied heavily on physical cash or lengthy payment procedures that are carried out during the procedure and after the consultation of the patients [5]. Instead, to streamline interactions between patients, healthcare facilities, and insurance providers, this revolutionary approach makes use of cutting-edge technologies, including mobile applications, digital wallets, and secure internet platforms [6,7]. This concept change, which embraces the digital era, aims to optimize the utilization of resources within the healthcare ecosystem and improve the overall patient experience [8,9]. The incorporation of technology has led to revolutionary innovations that continue to redefine the way people access and experience health services in the dynamic environment that comprises modern healthcare [10-12]. Cashless healthcare facilities have stood out among these significant advances as bringing about a new era of effectiveness, convenience, and improved patient care with the development of an economy without cash is consistent with international initiatives to update financial institutions and promote accountability [13]. These digital solutions offer previously unheard-of benefits to both healthcare professionals and patients by streamlining transactions, reducing administrative difficulties, and encouraging a cashless society [14-16]. The advantages of cashless hospital services have transformed the healthcare sector [17,18]. These tools are now essential elements of the modern medical scene, enhancing data security, facilitating transparent billing, and encouraging a patient-centered approach [19,20]. The potential of these changes holds to develop a healthcare environment that prioritizes availability, effectiveness, and flawless experiences and is beneficial for everyone [21,22]. By embracing medical transaction digitization, we show the way to a future where cashless hospital services are at the peak of patient-centric care and create the basis for a healthier and more connected society [23,24]. The objective of this article is to explore the advantages of cashless healthcare services to improve the efficiency and accessibility of healthcare.

## Review

Benefits of cashless services

**Figure 1:**
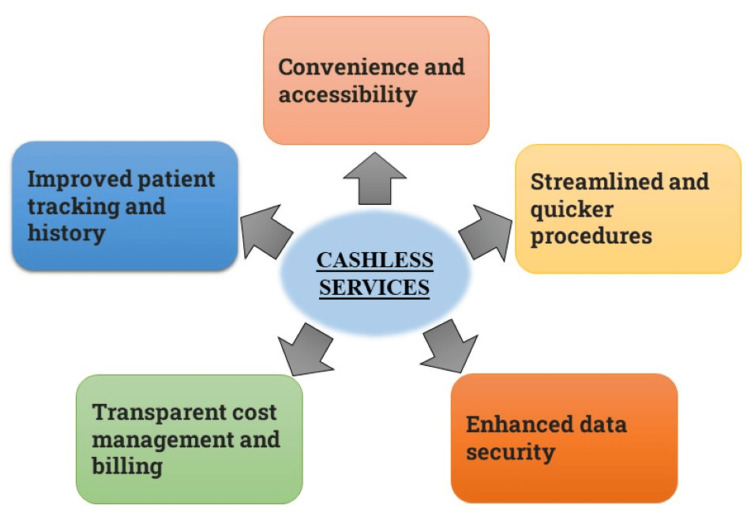
Features of cashless services Image created by Nikhil Sagare

Figure 1 shows the benefits of the cashless services applied in healthcare.

Convenience and accessibility

Cashless services are a preferred method of payment for both consumers and companies due to their convenience and accessibility. The availability of digital payment methods and mobile wallets has completely changed the way consumers conduct their financial transactions with the help of applications like Google Pay, Phone Pay, Paytm, and other digital internet banking methods [25]. One of the key advantages of cashless hospital services is the exceptional convenience and accessibility they offer. Patients no longer have to worry about carrying huge amounts of cash for medical procedures or consultations thanks to cashless transactions [26].

Patients may simply make payments using digital channels such as mobile payment applications, online payment gateways, or contactless payment cards, whether they are paying for medications, lab tests, or complicated surgeries [27]. Due to this accessibility, even people who live in distant places and may not have access to conventional banking facilities can still access vital healthcare services [28]. The broad use of cashless services is driven by ease and accessibility, which is changing the way people execute transactions and building a more effective and inclusive financial environment. The potential for more innovation and the incorporation of cashless services in several facets of daily life is enormous as technology develops [29,30].

Streamlined and quicker procedures

Cashless transactions drastically reduce administrative challenges for healthcare providers by eliminating the requirement for manual documentation [31]. Eliminating time-consuming manual processes is a crucial component of streamlining cashless transactions. People frequently experience delays when using traditional payment methods, such as cash or checks, as they have to count their money, wait for the checks to clear, or stand in line [6]. Quick electronic transactions are facilitated by cashless services, which drastically reduce processing times [32]. As the billing and payment process becomes digitized and streamlined, doctors and medical personnel can focus more on treating and caring for patients [33]. Furthermore, much faster insurance claim processing and verification processes result in quicker pay-outs and minimize the financial burden on patients [34,35].

Enhanced data security

Patients have good reason to be concerned about the security of their personal information in the era of data breaches and identity theft [36]. For the healthcare sector to protect sensitive financial and medical data, enhanced information security in cashless payment mechanisms is crucial. Patient data, such as healthcare records and financial information, is protected during transactions thanks to effective encryption measures. Unauthorized access is prevented by strict authentication mechanisms, such as two-factor authentication and biometric verification. Data protection is further strengthened by regular security audits and adherence to industry requirements [23]. Cashless payment techniques in healthcare promote smooth and safe financial transactions while protecting patient privacy by placing a high priority on data security. This inspires respect for patients, medical professionals, and insurance [37-39]. Cashless hospital services provide strong security precautions to protect sensitive data. These systems use multiple authentication methods to protect patient records, guaranteeing the privacy of sensitive medical data [40].

Transparent cost management and billing

One significant advantage of cashless healthcare services is the openness of the billing procedure [41]. Patients who want medical treatment are given detailed bills, which makes it easier for them to understand the breakdown of expenses [42]. Transparency empowers people to make informed decisions about their medical treatment and promotes trust between patients and healthcare professionals [43]. It gives organizations the ability to strategically allocate finances, maximize resource use, and improve operational effectiveness. It may improve financial transparency by offering itemized bills, real-time tracking, and frequent reporting. By enabling safe and auditable transactions and encouraging data-driven initiatives, cutting-edge technologies such as accounting programs and information analytics further increase transparency. Transparent cost control and billing are important business practices, but they are also essential components in building a strong image and promoting long-term success [44,45].

Improved patient tracking and history

In the healthcare sector, electronic health records (EHRs) and digital payment systems are routinely linked, allowing full patient tracking and management of health records [46]. Because of this unity, healthcare professionals can review patients' medical records, diagnoses, and treatment plans. As a result, by omitting unnecessary tests or treatments, clinicians are better equipped to provide customized, effective care [47,48]. This invention improves interprofessional communication, resulting in coordinated and effective care. To improve patient outcomes, patient monitoring and history systems are essential in emergency response, chronic disease management, and preventative care [49]. The incorporation of wearable technologies and data analytics improves patient insights as technology advances. Patient monitoring and history improvements have a substantial positive impact on healthcare quality, patient trust, and the advancement of medical research and practice [50,51].

Encouraging a cashless economy

Promoting a cashless environment in hospitals has several benefits both for patients and healthcare organizations. The use of digital payment systems improves transaction convenience and security in the healthcare industry. Patients no longer need to physically exchange currencies because they can easily settle healthcare expenses, consultation fees, and medication costs using smartphones or online platforms [52]. A cashless system reduces cleanliness problems, which are particularly important in hospital settings. Reducing physical contact with currency reduces the chance of spreading germs, in accordance with infection control procedures. Digital payment records simplify administrative procedures and increase hospital operational effectiveness [53]. Safe payment systems, user-friendly interfaces, and patient education are all necessary for the transition to cashless transactions in the healthcare industry. Hospitals can put the needs of their patients first, run more efficiently, and modernize financial transactions in the healthcare industry by adopting a cashless economy [54]. The healthcare services' increased measures to create a cashless society align with the progression toward a cashless approach. As more transactions move online because of the low use of physical cash, the whole financial ecosystem becomes more efficient. By reducing income tax fraud and money laundering, this move to digital finance fosters a more transparent and accountable financial sector [55].

Drawbacks

Table 1 shows the different challenges and drawbacks with their appropriate solutions regarding cashless services in the healthcare sector.

**Table 1:** Drawbacks of electronic systems in healthcare

Drawbacks	Description	Solution
Dependency on electronic systems	Risks in relying heavily on electronic systems for healthcare transactions, including technical issues, cyberattacks, and digital gaps. Impact on quality of care and potential interruptions in service due to power or internet outages [56].	Effective cybersecurity measures, user-friendly interfaces, and education for users [57].
Elderly and technologically challenged patients	Exclusion and challenges for elderly and technologically challenged individuals with cashless healthcare services. The complexity of electronic payment methods may hinder access to health services [58].	User-friendly interfaces, comprehensive support, and targeted user education to address technology disparities [58].
Fraud and cybersecurity risks	Vulnerability of patient data and transactions to fraud and cyber threats in cashless healthcare services. Data breaches, identity theft, and unauthorized access to medical records as consequences. Operational disruptions, loss of trust, and financial costs associated with breach incidents [59].	Requirement for strong cybersecurity measures and staff training [60].

Dependency on Electronic Systems

The disadvantage of heavily depending on electronic systems for financial transactions and patient records centers on the risks and difficulties that result from doing so in the context of cashless services in healthcare. Although electronic technologies are effective and convenient, there are hazards involved. Technical problems, software errors, or network problems might interfere with administrative and patient care procedures. The security of sensitive patient data is under threat due to the increased possibility of cyberattacks, which could result in privacy and confidentiality violations. The digital gap is further made clear by the fact that some groups, such as the elderly or those who are the least fortunate, may find it difficult to use these systems due to their limited access to or knowledge of technology [56]. The quality of care could be impacted if medical professionals and patients no longer connect personally. Power failures or Internet outages can cause additional interruptions in locations with fragile infrastructure. Healthcare providers must prioritize effective cybersecurity measures, create user-friendly interfaces, teach patients and employees, and maintain backup systems to provide uninterrupted services to overcome these downsides. Achieving successful integration requires striking a balance between the advantages of technological efficiency, security, and inclusivity [57].

Elderly and Technologically Challenged Patients

The disadvantage of cashless services in the healthcare industry for elderly and technologically impaired patients centers on the possible exclusion and challenges these people may face. While cashless services simplify transactions, they may unintentionally exclude parts of the population that are less proficient in technology. The complexity of electronic payment methods may be difficult for elderly people to understand since they are frequently less accustomed to digital platforms. This might cause dissatisfaction and prevent them from accessing health services. Similarly, those with limited technology skills who may struggle to adopt these systems owing to socioeconomic issues may exacerbate healthcare disparities. This flaw highlights the need for user-friendly interfaces and comprehensive support for these groups from healthcare providers and technology developers. To overcome the technology divide and deliver an improved, inclusive healthcare experience, targeted user education investments and alternative payment methods can be made. The healthcare industry can guarantee that improvements in cashless services benefit all demographics, supporting accessible and equitable healthcare delivery, and recognizing and solving these concerns [58].

Fraud and Cybersecurity Risks

The disadvantage of fraud and cybersecurity threats in the context of cashless healthcare services emphasizes how vulnerable patient data and financial transactions are to criminal activity. While the digitalization of transactions is convenient, it also creates opportunities for hackers to take advantage of flaws. Data breaches that violate patient privacy, cause identity theft, and allow unauthorized access to medical records frequently target healthcare systems that handle sensitive patient data [59]. Digital transactions are becoming more complicated, which opens the door to fraud as fraudsters develop sophisticated techniques to take advantage of system flaws. Breach incidents can cause major operational disruptions in the healthcare industry, affect patient trust, and cost patients and healthcare providers a lot of money. It is crucial to take strong cybersecurity precautions in order to reduce these threats. This involves implementing encryption mechanisms, conducting regular security audits, and training staff members to spot risks and take appropriate action [60].

## Conclusions

Cashless hospital services are revolutionizing the healthcare industry by enhancing convenience, security, and efficiency. Patients can concentrate on their physical and mental health without worrying about financial difficulties, which leads to less administrative effort, improved patient tracking, and transparent billing. Furthermore, the development of a cashless economy is consistent with international initiatives to update financial institutions and promote accountability. Cashless hospital services are expected to advance further as technology develops, piloting a new era of patient-centered healthcare. By embracing these revolutionary solutions, patients and healthcare providers can both look forward to a future with smooth, conveniently accessible, and outstanding healthcare services.
